# Quality of reporting and trends of emergency obstetric and neonatal care indicators: an analysis from Tanzania district health information system data between 2016 and 2020

**DOI:** 10.1186/s12884-023-06028-z

**Published:** 2023-10-07

**Authors:** Josephine Shabani, Honorati Masanja, Sophia Kagoye, Jacqueline Minja, Shraddha Bajaria, Yeromin Mlacha, Sia Msuya, Mahundi Masoud, Daudi Simba, Andrea B. Pembe, Ahmad Mohamed Makuwani, Habib Ismail, Maro Chacha, Claud Kumalija, Ties Boerma, Claudia Hanson

**Affiliations:** 1 Ifakara Health Institute, Dar es Salaam, Tanzania; 2 National Institute for Medical Research, Mwanza, Tanzania; 3 Kilimanjaro Christian Medical University College, Kilimanjaro, Tanzania; 4 University of Dar es Salaam, Dar-es-Salaam, Tanzania; 5 Muhimbili University of Health and Allied Sciences, Dar es Salaam, Tanzania; 6 Ministry of Health, Dodoma, Tanzania; 7 University of Manitoba, Manitoba, Canada; 8 London School of Hygiene & Tropical Medicine, London, UK

**Keywords:** Emergency obstetric care, District Health Information System, Obstetric complications, EmONC interventions, Institutional deliveries, Indicators

## Abstract

**Background:**

Routine health facility data provides the opportunity to monitor progress in quality and uptake of health care continuously. Our study aimed to assess the reliability and usefulness of emergency obstetric care data including temporal and regional variations over the past five years in Tanzania Mainland.

**Methods:**

Data were compiled from the routine monthly district reports compiled as part of the health management information systems for 2016–2020. Key indicators for maternal and neonatal care coverage, emergency obstetric and neonatal complications, and interventions indicators were computed. Assessment on reliability and consistency of reports was conducted and compared with annual rates and proportions over time, across the 26 regions in of Tanzania Mainland and by institutional delivery coverage.

**Results:**

Facility reporting was near complete with 98% in 2018–2020. Estimated population coverage of institutional births increased by 10% points from 71.2% to 2016 to 81.7% in 2020 in Tanzania Mainland, driven by increased use of dispensaries and health centres compared to hospitals. This trend was more pronounced in regions with lower institutional birth rates. The Caesarean section rate remained stable at around 10% of institutional births. Trends in the occurrence of complications such as antepartum haemorrhage, premature rupture of membranes, pre-eclampsia, eclampsia or post-partum bleeding were consistent over time but at low levels (1% of institutional births). Prophylactic uterotonics were provided to nearly all births while curative uterotonics were reported to be used in less than 10% of post-partum bleeding and retained placenta cases.

**Conclusion:**

Our results show a mixed picture in terms of usefulness of the District Health Information System(DHIS2) data. Key indicators of institutional delivery and Caesarean section rates were plausible and provide useful information on regional disparities and trends. However, obstetric complications and several interventions were underreported thus diminishing the usefulness of these data for monitoring. Further research is needed on why complications and interventions to address them are not documented reliably.

**Supplementary Information:**

The online version contains supplementary material available at 10.1186/s12884-023-06028-z.

## Introduction

Despite global efforts for achieving the Sustainable Development Goals of reducing maternal and neonatal mortality, deaths due to complications of pregnancy and childbirth remain to be high. In 2020, roughly 290,000 women died following pregnancy and childbirth with 94% of deaths occurred in low-middle income countries [1]. There has been substantial progress in child survival globally; still, in 2020, the number of neonatal deaths was 2.4 million with sub-Saharan Africa having the highest burden of neonatal mortality rate of 27 deaths per 1000 live births [2].

In Tanzania, maternal mortality continues to be high according to global estimates for 2020 at 238 maternal deaths per 100,000 live births [1]. On a similar note the neonatal deaths rate is estimated to be 20 per 1000 live births and the stillbirth rate stands at 19 deaths per 1000 births in 2020 [2].

Tanzania has prioritized Reproductive, Maternal, Newborn, Child & Adolescent Health (RMNCAH), which is demonstrated in various policies and national strategies such as One Plan III, National Five Year Development Plan (2021/2022–2025/2026) and the Health Sector Strategic Plan V(2021/22–2025/26) [3, 4]. The country has witnessed a progress in improved coverage and access of maternal care, especially through increased availability Emergency Obstetric and Newborn Care (EmONC) health facilities, institutional delivery (81%) and births attended by skilled health care providers (85%) [5, 6]. However, evidence indicate that there has been challenges in quality of care particularly in availability of competent health care providers and accessing EmONC services which are essential for reducing maternal and neonatal mortality [7–10].

The national RMNCAH plan for 2020–2025, called One Plan III, explicitly strategizes to provide universal access to quality delivery care [9, 11]. A key target is to maximize access to childbirth care in all health facilities, including hospitals, health centres and dispensaries. For example, the plan targets the upgrading of half of all health centres to provide comprehensive emergency obstetric care, this Caesarean section (C-section) which to a large extent had been achieved by 2020 [12].

Data to monitor progress and create accountability in countries is mostly derived from national representative household surveys, such as demographic and health surveys which are generally conducted once every five years or so [13]. Routine health information systems are, however, increasingly recognized as an important potential source to steer national and sub-national strategies and policies [14, 15]. The introduction of the District Health Information System (DHIS2), and the move from paper-based to hybrid systems, with district level computerization and link to a web-based system [16], provides greater opportunities to analyze data up to facility level [17]. The Tanzania DHIS2 has incorporated several EmONC indicators in the continuous routine facility-based data collection which presents unique opportunity for analysis.

The aim of this study is to assess the capacity of EmONC interventions during childbirth in Tanzania. Specifically, using DHIS2 data, the study will describe the changes in provision of EmONC services over the past five years (2016–2020) and noted variations across regions. The study further aims to review consistency of reporting of complications and variations in obstetric interventions by region to hence provide a better understanding of the quality of data.

## Methods

### Study design and setting

We used cross-sectional data from 2016 to 2020 which is collected as part of routine health information system data. Since 2020, Tanzania classifies as a lower middle-income county with a total population of 61 million people [18]. The health system is this country is organised as a district health system with a strong focus on primary health care since independence [19]. The health system is structured in a three-tier pyramid, with primary level facilities; level 1 includes (i) community, (ii) dispensary, the lowest physical structure servicing one or few villages, (iii) health centre, some of being designated as Comprehensive EmONC health facilities and (iv) district hospital or a designated district hospital located at a district council. Level 2 involves Regional Referral Hospitals (RRH) which function as referral hospitals and provide specialist medical care. Level 3 includes Zonal and National Hospitals which offer advanced medical care and are teaching hospitals for medical, paramedical, and nursing training [20, 21].

### Data source

This study is based on a secondary analysis of routine health facility data available through the Tanzania Mainland District Health Information System 2 (DHIS2) database. Since 2013, the health management information system in Tanzania has been operating as a hybrid digitalised system. Facility-based recording of events is done in paper-based registers which is later summarised using tally and summary sheets and then monthly aggregated data on events including the number of births, complications and interventions are produced [17]. The monthly summary sheets are sent to the district, and DHIS2 focal person enters the aggregated service statistic data into a web application which is part of the DHIS2 system. Faith-based and other private facilities are supposed to report to the DHIS2, although private-for-profit facilities have reservations with regulations. By 2020, Tanzania had a total of 8,458 health facilities comprising of 369 hospitals, 926 health centres and 7,163 dispensaries [22].

### Data analysis

All indicators in this analysis are documented on the labour and delivery monthly summary form (DHIS2 Register Form 12). This form documents the number of all facility birth, the mode of delivery, as well as counts of complications seen at the health facilities and counts of EmONC interventions managed in the facilities.

We extracted data from all levels of health care in Tanzania which is aggregated at district level and summarized counts for institutional births, C-section, antepartum, intrapartum and postpartum complications from the DHIS2 database, as well as EmONC interventions for the period 2016–2020 and aggregated these data to the regional and Tanzania Mainland levels for our analyses (Table 1). All counts were extracted into Excel. Stata 17 software was used for the analysis.

**Table 1:** Coverage of births in the population, mortality rates, place of delivery and mode of delivery by year, DHIS2 data, Mainland Tanzania, 2016–2020

	2016	2017	2018	2019	2020
**Institutional deliveries (estimated population coverage)**					
Facility deliveries, reported (N)	1,284,356	1,426,434	1,675,409	1,795,109	1,881,194
Institutional deliveries, adjusted for missing reports and extreme outliers (N)	1,393,380	1,520,218	1,751,180	1,865,101	1,942,116
C-sections (N)	138,204	152,445	166,275	178,924	192,777
Districts reporting rate (delivery forms) (%)	96.1	96.8	97.6	98.5	98.0
Total deliveries in population (estimated) (N)	1,957,937	2,071,584	2,237,029	2,298,388	2,375,811
Population coverage institutional deliveries (%)	71.2	73.4	78.3	81.1	81.7
Population coverage C-sections per 100 deliveries (%)	7.1	7.4	7.4	7.8	8.1
**Institutional mortality**					
Stillbirths per 1,000 facility births	15.9	14.3	11.7	10.6	10.0
Fresh stillbirths per 1,000 facility births	6.7	6.2	5.1	4.5	4.3
Percent fresh among all stillbirths	42.1	43.3	43.4	42.9	43.2
Maternal mortality per 100,000 births	75.7	69.6	56.0	56.3	54.0
**Place of delivery (distribution of institutional deliveries)**					
Hospital	44.3	41.2	34.7	32.5	28.3
Health centre	23.3	25.3	26.2	27.3	30.5
Dispensaries	32.3	33.4	39.1	40.2	41.2
Total	100.0	100.0	100.0	100.0	100.0
**Mode of delivery (distribution of institutional deliveries)**					
Spontaneous vaginal birth	88.7	88.6	89.3	89.2	88.9
Vacuum extraction	0.4	0.4	0.3	0.3	0.3
Breech	0.8	0.8	0.7	0.6	0.7
C-section	10.2	10.2	9.6	9.8	10.1
Total	100.0	100.0	100.0	100.0	100.0

Population estimates for coverage of deliveries (childbirth event) and births (live and stillbirths) by health facilities and C-section were obtained using methods described elsewhere [23–25]. In brief, we adjusted the number of events (numerators) by taking into account the health facility reporting rates by assuming that non-reporting facilities do provide services but only at a lower rate than reporting health facilities (one-fourth of reporting facilities), in line with other Tanzania facility data analyses [26]. Since Tanzania’s population projections did not give plausible results (the last census was in 2012), health facility data were used to derive denominator using a method based on the number of first antenatal care (ANC) visits (ANC1) [27]. The denominator *number of deliveries* in the population was based on the reported number of first ANC visits. In Tanzania first ANC visit coverage is very high in all regions; 98% according to the TDHS 2016 [28]. We adjusted the number by adding 2-percent of non-users and subtracted abortions occurring after the first ANC visit (median about 20 weeks, assumed 3% loss) for *estimated deliveries*. To obtain estimated births we added multiple births for (1.5%) [29]. Thus, the term deliveries were used to describe live and stillbirth per childbirth event while the term births encompassed all babies born including multiples.

To assess the appropriateness health facility, denominator was derived to compare the indicators of institutional delivery rate and C-section using the 2016 facility-data derived denominator constructed indicators with the DHS 2015/16 [28] estimates. For delivery complications and intervention coverage, the reported numbers of institutional deliveries were considered as denominators and therefore no adjustments were made. In addition, a computation of institutional stillbirth and maternal deaths statistics from the reported data within DHIS2 was done.

Subnational variation in key indicators was examined for the 26 regions of the Tanzania Mainland, based on the aggregated district data. To synthesize the differences, the mean value for the three regions with the highest and the three the lowest values was computed as an indicator, for all five years combined. The incidence of complications and intervention coverage were assessed whether if there was association with the coverage for institutional deliveries per population level that grouped regions into low coverage (< 70%, 6 regions), middle coverage (70–79%, 12 regions) and high coverage ( > = 80%, 8 regions) during 2016–2020. The cut-offs were chosen pragmatically to reflect the variation observed. The proportion of complications documented, and interventions disaggregated by health facility levels (i.e. dispensary, health centre and hospital) was also done.

### Ethical consideration

Permission to use the data was received from the Ministry of Health (MOH) and ethical clearance from the National Institute for Medical Research (NIMR/HQ/R.8a/VoL.IX/4099). Take note that DHIS2 data are routinely collected service statistics hence no individual consent was required.

## Results

The DHIS2 summary district reporting was over 95% throughout 2016–2020 and near complete with 98% during the last three years (Table 1).

**Fig. 1:**
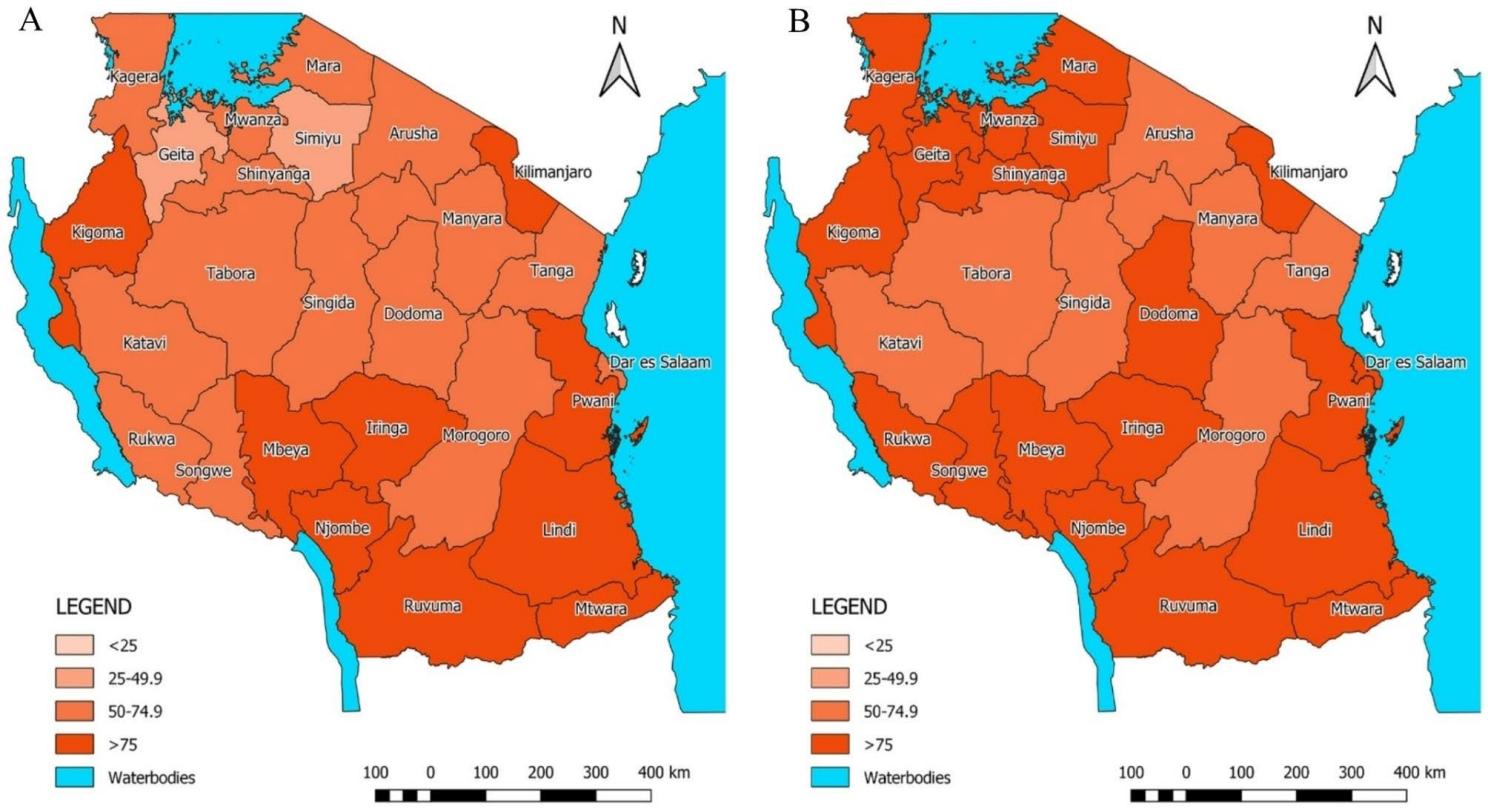
Facility delivery coverage (**A**) 2016 and (**B**) 2020, DHIS2 data

**Fig. 2:**
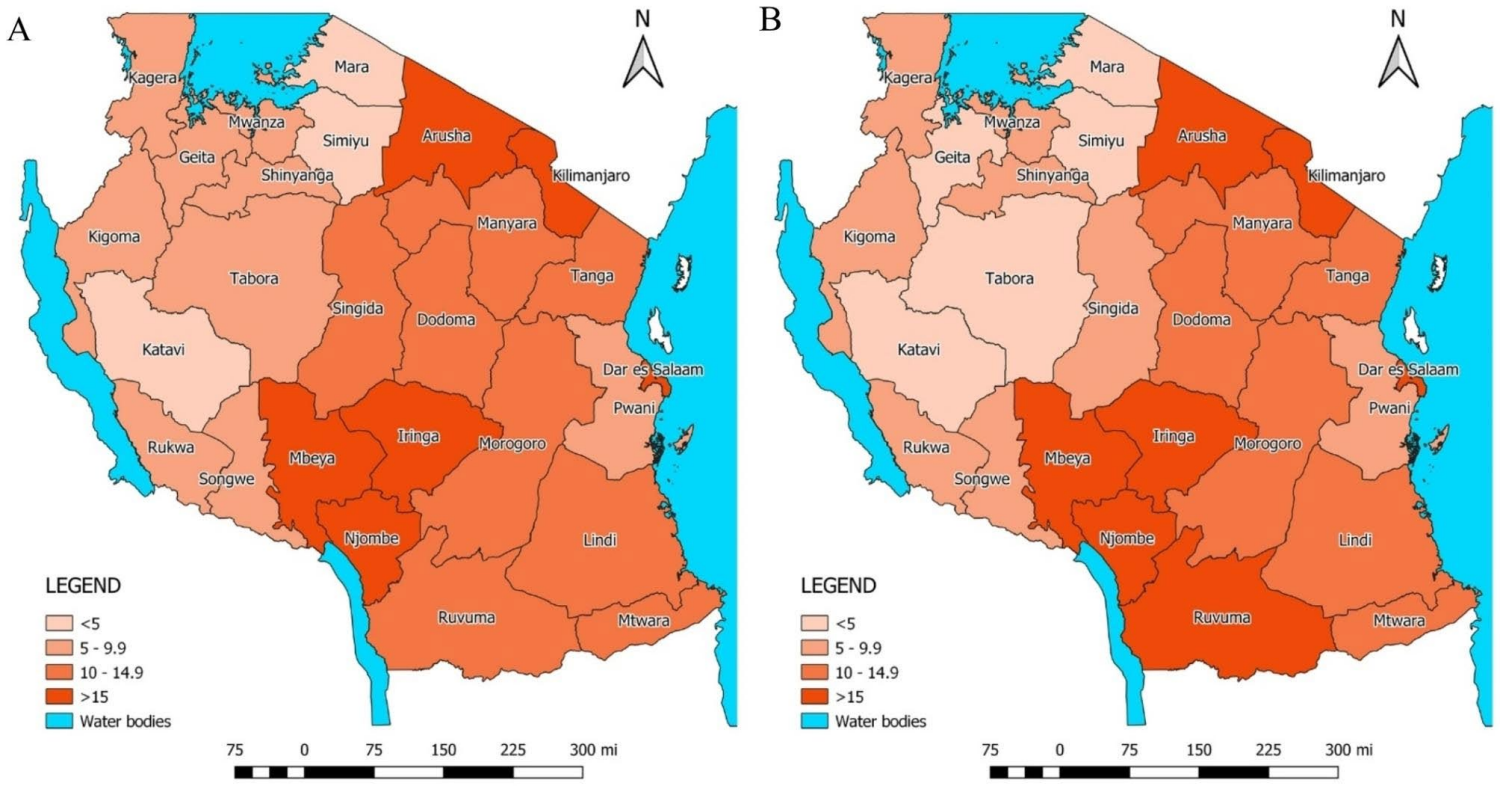
Caesarean Section rate (per institutional delivery) in (**A**) 2016 and (**B**) 2020, DHIS2 data

Reported institutional deliveries increased from 1,284,356 in 2016 to 1,881,194 deliveries in 2020. The number of institutional deliveries adjusted for underreporting and outlier increased from nearly 1.4 million in 2016 to over 1.9 million in 2020. The estimated population coverage of institutional deliveries increased from 71.2% to 2016 to 81.7% in 2020 (Table 1). By 2020, institutional deliveries coverage ranged from a low of 64% in Manyara and Katavi regions to over 90% in Iringa and Njombe (Fig. 1). C-section rates in the Tanzania Mainland also increased from 6.9 to 8.0% during 2016–2020, driven by the increase in institutional deliveries. Regional variation in C-section rates was large and persistent over time, ranging from less than 3% in Simiyu, Tabora, Katavi and Geita, to over 15% in Njombe, Dar es Salaam, Iringa, Mbeya, Arusha and Kilimanjaro (Fig. 2, Annex Table 1).

**Fig. 3:**
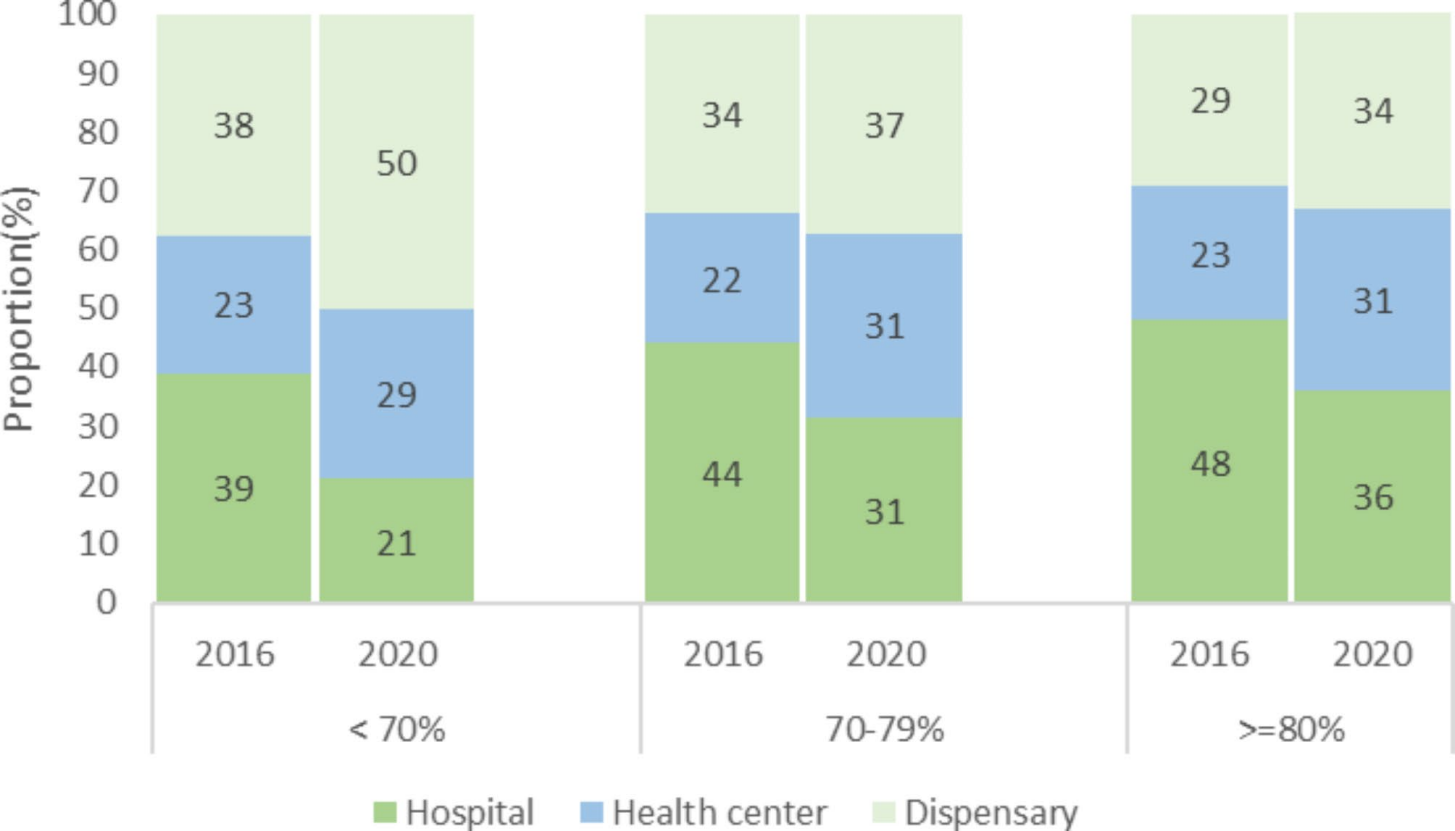
Distribution of institutional delivery by region, grouped according to delivery coverage in 2016 (< 70%, 70–79% and 80% and over)

The major increase in institutional delivery coverage was driven by an increase of births taking place in lower-level health facilities. Whereas the proportion of deliveries recorded in dispensaries and health centres increased consistently between 2016 and 2020, the opposite is observed in hospital deliveries. (Table 1; Fig. 3). The largest increase in facility delivery was seen in dispensaries in regions where institutional birth rate was below 70% (from 38 to 50%), in settings with an institutional birth rate between 70 and 80%, we observed an increase in health centres birth from 22 to 31% within the five years (Fig. 3).

Among institutional deliveries, stillbirth rates declined from 16.9 to 10.0 per 1000 during 2016 to 2020, with fresh (intra-partum) stillbirths accounting for about 42–43% of all stillbirths in all years. Maternal death reporting was at 54–76 per 100,000 births 2016–2020. Hospitals reported highest proportion of maternal deaths (41.4%), followed by dispensaries (33.1%) and health centres (25.1%) over 5 years (2016–2020).

Assisted breech delivery was reported to be 0.7% of deliveries with little change over time (Table 1). Data showed vacuum delivery was uncommon, and C-section rates were roughly 10% of institutional deliveries with only minor variations throughout the years.

Reporting of complications was consistent throughout the period of five years (Table 2). Complications before birth including antepartum haemorrhage, premature rupture of membranes, pre-eclampsia and HIV/AIDS stage III or IV were all reported by less than 1% of all deliveries. Combining all three clinical presentations, hypertensive disorders were reported for 1.0-1.4% of all deliveries. Anaemia and malaria were more frequently reported. Postpartum bleeding was reported to complicate just under 1% of institutional births between 2016 and 2020 (Table 2, Annex Table 1).

**Table 2:** Institutional complications and interventions by timing of complication and by year (DHIS-2 data, Tanzania 2016–2020)

	2016	2017	2018	2019	2020
Facility deliveries, reported (N)	1,284,356	1,426,434	1,675,409	1,795,109	1,881,194
**Antepartum complications**					
Antepartum hemorrhage	0.4	0.4	0.3	0.3	0.3
Premature rupture of membranes	0.6	0.6	0.5	0.5	0.5
Anemia	1.1	1.3	1.4	1.8	1.1
Malaria	8.2	8.7	8.5	8.0	9.5
HIV (Stage III or IV)	0.3	0.3	0.3	0.3	0.3
Hypertensive disorders of pregnancy (total)	1.3	1.4	1.1	1.1	1.0
Pregnancy induced hypertension	0.6	0.6	0.6	0.5	0.4
Pre-eclampsia	0.5	0.5	0.4	0.4	0.4
Eclampsia	0.3	0.3	0.2	0.2	0.2
**During / after delivery complications**					
Postpartum hemorrhage (PPH)	0.8	0.7	0.7	0.7	0.7
Obstructed labour	2.3	2.2	1.9	1.9	1.8
Retained placenta	0.2	0.2	0.2	0.2	0.1
Third degree tear	0.1	0.1	0.0	0.0	0.0
Ruptured uterus	0.1	0.1	0.1	0.1	0.1
Pre-eclampsia	0.7	0.7	0.5	0.6	0.6
Eclampsia	0.5	0.5	0.4	0.4	0.4
Sepsis	0.2	0.1	0.1	0.1	0.1
**Interventions**					
Antibiotics given	10.1	9.9	8.8	8.8	8.8
Oxytocin administered (AMTSL)	94.0	95.6	95.3	97.0	90.6
Magnesium sulphate: given per 100*	36.8	35.0	38.2	36.6	35.6
Uterotonics for complications^	17.8	12.1	9.4	8.6	8.7
C-section per 100 institutional deliveries	10.2	10.2	9.6	9.7	10.1
Total	100.0	100.0	100.0	100.0	100.0

*cases of pre-eclampsia and eclampsia; ^PPH and retained placenta

Antibiotics were provided for almost 10% of institutional deliveries. Use of preventive uterotonics as part of Active Management of the Third Stage of Labour (AMTSL) was recorded for over 90% of institutional deliveries. The reported use of curative uterotonics for treatment was low. The ratio of reported numbers of women given uterotonics for treatment of reported cases of postpartum bleeding or retained placenta was as low as 9% during 2018–2019. Magnesium-Sulphate was provided to just over one-third of cases according to the ratio of reported treatments to reported cases with hypertensive disorders of pregnancy (pre-eclampsia or eclampsia). We observed a limited variation with regard to reports of complications and provision of interventions by overall institutional deliveries rate (Table 3).

**Table 3:** Summary of regional statistics for key complications and interventions, 2016–2020 combined, mean of the three highest and three lowest value regions and by population coverage institutional births

	Mainland	Mean of 3 regions			Regional coverage inst. births		
	Mean 2016–2020	highest 3	lowest 3		< 70%	70–79%	>=80%
**Pre-delivery**							
Malaria in pregnancy	8.6	21.7	0.5		8.9	8.7	7.2
Hypertensive disorders	1.2	2.7	0.4		0.8	1.2	1.3
Breech	0.7	1.1	0.5		0.6	0.7	0.8
**During / after**							
PPH	0.7	1.3	0.4		0.6	0.7	0.8
Obstructed labour	2.0	3.5	0.8		1.3	2.3	2.4
(Pre-) Eclampsia	1.0	2.3	0.3		0.7	1.0	1.2
**Interventions**							
Oxytocin	94.5	98.5	90.0		94.8	96.3	94.2
Uterotonics	11.3	27.5	2.7		9.5	13.0	9.7
Magnesium sulphate	36.4	49.6	27.5		36.4	37.4	37.1
C-section	10.0	22.4	3.2		6.6	10.7	13.6

High proportion of complications and interventions was reported at hospital level. There was also an increase in reporting of complications after delivery at health centres. Hypertensive disorders were highly reported by hospitals in 2020(47.7%) compared to 38.1% in 2016. The highest reported EMONC intervention among all health facility levels was oxytocin (74.2%) followed by magnesium sulphate at hospital level (28.7%) (Annex Table 2).

## Discussion

DHSI2 data on childbirth care have been used sparingly, even though maternity registers are potentially a rich source of information for planning and programming. Our analysis of five years of national and regional data shows the utility and current limitations of health facility data despite the fact that facility reporting was near 100%. Numbers of events, complications and interventions showed a good consistency over time with few outliers.

Estimated population coverage of institutional deliveries and C-sections show good consistency with the levels and trends observed in national surveys where the latest preliminary findings from Demographic and Health Survey 2022 suggest that 81% of births take place in facilities in 2020 and 2021 [30]. Comparing the regional institutional deliveries for 2016 with the DHS 2015/16 and DHS 2022/23 with DHIS2 data, indicates reasonable correlation (Annex Fig. 1A & Annex Fig. 1B) Similarly, a reasonable correlation is observed in relation to the C-section rates (Annex Fig. 2).

The analysis provides important up-to-date information on the recent preferences of the place of childbirth care. The Tanzanian policy of upgrading health centres to every ward and provision of dispensary at village level is likely to have led to the increase in utilization during 2016–2020. The C-sections rate using approximated births based on adjusted ANC-1 data revealed lower rates compared with DHS 2015/16 rates – with increasing discrepancies in places where the rates were high (Annex Fig. 1A & Annex Fig. 1B). Data from the DHS 2023 are still pending.

Our analysis indicated an acceleration of the trend towards childbirth care in lower-level health facilities (dispensaries and health centres). The observed trend is likely to reflect the major investments which the Tanzanian Government has made to improve the availability of EmONC services in health centres [4, 31]. However, the trend of increased deliveries in dispensaries may be a concern as these facilities are not able to offer the same higher quality-of-care and advanced management of complications as hospitals [32–34]. A similar trend of use of lower-level health facilities has recently been described based on DHS data for the period prior to 2016 [6, 21].

The data on mortality are less assuring in terms of data quality – even considering that health facility and population-based estimates are not fully comparable. The DHIS2 reported institutional stillbirth rates are 10.0 deaths per 1,000 facility birth (2020) while international, population-based estimates report suggest rates around 18 deaths per 1000 births [2]. The DHIS2 reported institutional maternal mortality ratios is 54 per 100,000 birth (2020) while the latest international estimates propose a ratio of 238 for 2020 (uncertainty interval 174–381) [1].

Limitations are seen in the reporting of obstetric complications, although comparative estimates from sub-Saharan Africa are not available for all complications. Our DHIS2 analysis showed a rate of assisted vaginal breech of around 0.8%. This is lower than estimates by the WHO multi-country survey carried out in 21 countries including 287 referral facilities, suggested a prevalence of breech presentation of 4% [35]. The estimate of 4% is in line with global estimates proposing that 3–5% of term birth are breech presentation [36]. While our data do not allow a disaggregation of breech by mode of delivery, it seems unlikely that about 75% of babies in breech position are delivered by C-section in Tanzania. A study from the national referral hospital in Dar-es-Salaam suggested that little below 50% of women presenting with breech were delivered by C-Sect. [37] but this estimate may not depict the situation in Mainland Tanzania outside the cities.

Further, our analysis suggests that less than 0.5% of births are by vacuum extraction. Vacuum extraction rates depend on medical customs and vary in the European Union between 0.5% in Romania to 15.1% in Ireland [38]. In Africa, the use of vacuum extraction is largely limited to larger hospitals and reported rates in hospitals are often below 1% [39]. In Tanzania, vacuum extraction is only done at hospitals and selected health centres further explaining the low rate.

The interpretation of the reports on complications is constrained as the estimates are not plausible. Antepartum bleeding is estimated to occur in between 2 and 5% of all pregnancies globally [40], while the facility reports suggests that it is only complicating 0.3% of institutional deliveries in Tanzania. Global data suggest that premature rupture of membranes complicates about 3% of births [41], however, this condition is only recorded for 0.5% of births in our study. Typically, 5–10% of pregnancies are complicated by hypertensive disorders globally, including gestational hypertension, pre-eclampsia, and eclampsia with about 2–4% developing pre-eclampsia [42, 43]. The health facility reports suggest lower rates of 0.3% of each, hypertensive disorders, and pre-eclampsia. Postpartum Haemorrhage (PPH) may complicate around 5% of birth [44] and our DHIS2 analysis showed a rate below 1%.

Our study reports a similar picture for reports of the management of complications. It is expected that almost all cases PPH or retained placenta were treated with curative oxytocin but the DHIS2 report a rate below 10%. It is to note that PPH is reported to be the leading cause of maternal deaths in Tanzania, the condition is estimated to cause roughly one-third on maternal deaths [45, 46].

It was also expected that near 100% of women with pre-eclampsia or eclampsia received Magnesium-sulphate injection as these patients would have benefited from this treatment assuming that only cases of severe pre-eclampsia and eclampsia are recorded. Again, it is to note that 18.9% of maternal deaths are due to hypertensive disorders [46].

The low reporting of complications and interventions addressed to complications is a concern. However, our analysis does not provide any explanation if (i) only the documentation was omitted or (ii) if coverage rates for treating complications are indeed low. Limited numbers and capacity of health care providers and lack of diagnostic options may complicate the identification of complication. In view of the human resource shortages, underreporting of obstetric complications has also been described even during special studies such as described by a near-miss study in Tanzania – albeit to a lesser extent [47]. This near-miss study reported, however, that obstetric complications were in fact mostly appropriately treated [47]. The present strong investment into the human resources in the country, particular in higher trained nurses and doctors may however change the situation in the near future [48].

The latest National Service Availability and Readiness Assessments (SARA) assessing health facility readiness suggest that 95% of health facilities have a blood pressure machines to detect hypertension. Little above 50% can test for protein in the urine to confirm pre-eclampsia. Little over 50% of health facilities report that they offer the package of vacuum extraction, manual removal of the placenta, parental administration of antibiotics, parental administration of anti-convulsant, and parental administration of uterotonics in 2020, with similar levels throughout the past 5 years [31, 49].

The low reporting may thus only be in parts explained by missing facility readiness. Recent studies in validating of HMIS data for newborn health have pointed to low validity of some indicators. Underlying barriers include the register design, lack of training and supervision, and providers who feel to prioritise clinical care at the expense of documentation [50, 51]. An additional underlying reason might be that nurses and midwives prioritise clinical case notes but not the DHIS2 reports [52].Human resource for health shortage (52%) [53] both in numbers and capacity add stress on frontline workers. Moreover, they have additional tasks like cleaning, planning, patient care, patient transfer, and a variety of other competing clinical and non-clinical priorities and multiple registers to fill (8 for Prevention of Mother To Child Transmission (PMTCT) alone, and additional for any research or vertical program). This may have reduced the time and ability to consistently fill the DHIS2 forms.

Analysis from this study has both strengths and weaknesses. We included district reports summarising childbirth care in Tanzania Mainland over five years, more than 96% of the districts reported on key maternal and newborn indicators over five years. Health facility reporting in Tanzania includes public and some private facilities; thus, data are reasonably representative of national service statistics. Our quality check indicated good numerical completeness and consistency. Weaknesses lie in the fact that documentation of complication and intervention addressing the complication maybe incomplete. Our analysis is constraint by the lack of population and facility-based complication rates from sub-Saharan Africa; global estimates may not necessarily be applicable to high fertility settings.

## Conclusion

In summary, we see a mixed picture in terms of usefulness of the DHSI2 data. The information of trends of place of delivery and the C-section rates seems plausible and are relevant – supporting the potential use of facility and DHIS2 data. The data suggested an improvement in institutional delivery by 10% points from 71.2 to 2016 to 80.7% in 2020 in Tanzania Mainland with large increases in lower-level health facilities. The C-section remained stable at around 10%.

However, the data on mortality as well as obstetric complications and interventions to address them seemed to be incomplete, thus limiting their usefulness for monitoring. Research is needed to understand if the underlying reasons are rooted in (i) sub-standard clinical knowledge, (ii) missing diagnostic devises, (iii) lack of documentation in case notes, (iv) forgotten transfer of clinical findings from case notes to DHIS2, or (v) errors in aggregation for the summary reports. The low rate of stillbirth reporting is another concern needing targeted data quality assessment for maternal and newborn indicators in DHIS2 and probably a comprehensive intervention also addressing blame and shame. In view of the questionable information obtained, one may consider reducing the present number of EMONC indicators to those most essential under the assumption that reporting fewer indicators will increase the quality. Moving to electronic perinatal e-registers may support improved reporting and data quality [54]. Nevertheless, data improvement strategies need to be implemented together with structural improvement in number and quality of staff to ensure recognition of complications and sufficient time for documentation.

In addition, there is a need to establish reference data on obstetric complications specific for Sub-Saharan Africa in view that high fertility high and the continent has a different underlying pattern of communicable and non-communicable diseases.

### Electronic supplementary material

Below is the link to the electronic supplementary material.

Supplementary Material 1

## Data Availability

The main data source is the routine health information system. Data are reported by health facilities to district offices on a monthly basis using standardised reporting forms. At the district offices, data is entered into computers using District Health Information System software version 2 (DHIS2) https://dhis2.org. In the DHIS2, data can be summarised and aggregated into district, regional, zonal and national levels.

## Abbreviations

(RMNCAH): Reproductive, Maternal, Newborn, Child & Adolescent Health.
(EmONC): emergency and neonatal obstetric care.
(DHIS2): District Health Information System.
(C-Section): Cesarean Section.
(ANC): Antenatal care visits.
(PPH): Postpartum bleeding.
