# Supplementary material for: Quality of reporting and trends of emergency obstetric and neonatal care indicators: an analysis from Tanzania district health information system data between 2016 and 2020
Source: BMC Pregnancy Childbirth. 2023 Oct 7;23:716. doi: 10.1186/s12884-023-06028-z (PMC10559477; doi:10.1186/s12884-023-06028-z)
Supplement: Supplementary file 1 — Supplementary Material 1 [file 12884_2023_6028_MOESM1_ESM.docx]

Annex

Figure 1: Regional institutional deliveries (%) comparing (A) DHIS2 and DHS 2015&16 and (B) DHIS2 and DHS 2022/23 data

Figure 2: Regional C-section rates comparing DHIS2 and DHS 2015&16 data

*Table 1:* *Summary of regional statistics for key complications and interventions, 2016-2020 combined*

|  |  | | |  |  | | |  |  | | | |
| --- | --- | --- | --- | --- | --- | --- | --- | --- | --- | --- | --- | --- |
|  | Pre-delivery | | |  | During/after | | |  | Interventions | | | |
| Region | Malaria | hypertensive disorders | Breech |  | PPH | Obstructed labour | (Pre-) eclampsia |  | Oxytocin | Uterotonics | Magnesium sulphate | C-section |
| Arusha | 0.4 | 1.7 | 0.6 |  | 1.0 | 1.8 | 0.8 |  | 96.0 | 30.5 | 25.7 | 14.3 |
| Dar es Salaam | 1.6 | 4.2 | 1.2 |  | 1.1 | 2.7 | 1.8 |  | 91.2 | 22.5 | 43.0 | 21.3 |
| Dodoma | 1.3 | 0.7 | 0.6 |  | 0.8 | 2.1 | 0.4 |  | 94.7 | 18.2 | 29.9 | 10.3 |
| Geita | 21.4 | 0.5 | 0.7 |  | 0.5 | 1.1 | 0.2 |  | 96.7 | 3.8 | 36.6 | 4.0 |
| Iringa | 0.8 | 1.1 | 0.7 |  | 0.4 | 3.0 | 0.5 |  | 96.3 | 7.4 | 36.1 | 19.2 |
| Kagera | 14.9 | 0.5 | 0.5 |  | 0.5 | 1.8 | 0.3 |  | 96.2 | 3.3 | 31.4 | 8.1 |
| Katavi | 11.7 | 0.5 | 0.4 |  | 0.4 | 0.7 | 0.2 |  | 91.7 | 9.2 | 49.8 | 3.0 |
| Kigoma | 12.6 | 0.4 | 0.9 |  | 0.8 | 1.6 | 0.2 |  | 95.6 | 5.2 | 39.6 | 5.2 |
| Kilimanjaro | 0.4 | 1.7 | 0.6 |  | 1.1 | 2.8 | 1.7 |  | 96.7 | 8.6 | 32.6 | 17.2 |
| Lindi | 21.3 | 2.0 | 1.1 |  | 1.3 | 2.6 | 0.8 |  | 95.3 | 6.8 | 34.7 | 11.4 |
| Manyara | 0.7 | 0.7 | 0.8 |  | 0.8 | 2.3 | 0.4 |  | 94.9 | 3.0 | 30.8 | 11.4 |
| Mara | 13.4 | 0.4 | 0.6 |  | 0.7 | 2.2 | 0.2 |  | 97.2 | 2.2 | 42.5 | 4.7 |
| Mbeya | 3.9 | 1.4 | 0.7 |  | 0.7 | 4.2 | 0.5 |  | 97.1 | 29.7 | 38.3 | 17.3 |
| Morogoro | 12.0 | 1.8 | 0.9 |  | 1.0 | 2.7 | 1.1 |  | 98.6 | 8.4 | 33.3 | 12.3 |
| Mtwara | 22.3 | 1.7 | 0.8 |  | 1.3 | 3.2 | 0.7 |  | 96.4 | 11.5 | 37.4 | 12.3 |
| Mwanza | 7.7 | 1.3 | 0.6 |  | 0.7 | 1.0 | 0.9 |  | 94.5 | 8.7 | 30.9 | 6.4 |
| Njombe | 0.9 | 0.6 | 0.7 |  | 0.5 | 2.9 | 0.3 |  | 87.2 | 17.6 | 41.8 | 26.6 |
| Pwani | 6.7 | 1.7 | 0.8 |  | 0.9 | 2.6 | 0.8 |  | 93.9 | 13.5 | 44.2 | 9.5 |
| Rukwa | 5.7 | 0.3 | 0.5 |  | 0.4 | 1.7 | 0.2 |  | 99.0 | 9.4 | 49.3 | 5.6 |
| Ruvuma | 11.0 | 0.9 | 0.6 |  | 0.7 | 3.2 | 0.4 |  | 93.7 | 17.9 | 37.3 | 15.7 |
| Shinyanga | 12.4 | 0.9 | 0.7 |  | 0.5 | 1.0 | 0.5 |  | 96.4 | 11.5 | 33.7 | 5.3 |
| Simiyu | 5.9 | 0.4 | 0.5 |  | 0.5 | 0.8 | 0.1 |  | 94.0 | 6.5 | 38.1 | 2.8 |
| Singida | 2.9 | 0.6 | 0.6 |  | 0.6 | 1.3 | 0.4 |  | 98.1 | 7.1 | 26.8 | 9.3 |
| Songwe | 3.2 | 0.5 | 0.7 |  | 0.5 | 2.3 | 0.2 |  | 96.5 | 14.2 | 49.6 | 7.4 |
| Tabora | 13.1 | 0.7 | 0.7 |  | 0.7 | 1.0 | 0.3 |  | 95.4 | 4.0 | 37.2 | 4.2 |
| Tanga | 8.7 | 1.1 | 0.8 |  | 0.6 | 2.4 | 0.6 |  | 95.2 | 15.2 | 35.0 | 10.0 |
| National | 8.6 | 1.2 | 0.7 |  | 0.7 | 2.0 | 0.6 |  | 95.4 | 11.3 | 36.4 | 10.0 |

*Table 2:* *Summary of statistics for key complications and interventions, 2016 and 2020 by health facility level*

|  | Hospital | |  | Health centre | |  | Dispensary | |
| --- | --- | --- | --- | --- | --- | --- | --- | --- |
|  | 2016 | 2020 |  | 2016 | 2020 |  | 2016 | 2020 |
| **Pre-delivery** |  |  |  |  |  |  |  |  |
| Malaria in pregnancy | 10.3 | 10.2 |  | 22.1 | 7.1 |  | 45.9 | 2.3 |
| Hypertensive disorders | 38.1 | 47.7 |  | 22.0 | 38.2 |  | 4.3 | 14.5 |
| Breech | 10.5 | 9.6 |  | 14.7 | 15.9 |  | 13.4 | 22.1 |
| **During / after** |  |  |  |  |  |  |  |  |
| PPH | 0.2 | 20.6 |  | 0.4 | 28.9 |  | 0.1 | 28 |
| Obstructed labour | 20.8 | 15.8 |  | 29.4 | 13.0 |  | 30.2 | 11 |
| (Pre-) Eclampsia | 33.1 | 29.4 |  | 25.5 | 31.8 |  | 17.3 | 44.4 |
| **Interventions** |  |  |  |  |  |  |  |  |
| Oxytocin | 27.2 | 23.1 |  | 48.9 | 21.7 |  | 74.2 | 13.6 |
| Uterotonics | 25.2 | 27.2 |  | 26.8 | 20.2 |  | 20.5 | 1.2 |
| Magnesium sulphate | 21.9 | 28.7 |  | 13.6 | 42.4 |  | 3.1 | 81.8 |
| C-section | 25.6 | 20.9 |  | 10.7 | 15.8 |  | 2.3 | 3.3 |
